# Managing a Case of a Congenital Cystic Eyeball: Case Report with Review of Literature

**DOI:** 10.1155/2022/3945537

**Published:** 2022-05-23

**Authors:** Aashish Raj Pant, Rinkal Suwal, Purushottam Joshi, Santosh Chaudhary

**Affiliations:** ^1^Department of Oculofacial Plastic Surgery, Mechi Eye Hospital, Jhapa, Nepal; ^2^Department of Optometry, B.P. Eye Foundation, Hospital for Children, Eye, ENT and Rehabilitation Services (CHEERS), Bhaktapur, Nepal; ^3^Department of Vitreo-Retina, Mechi Eye Hospital, Jhapa, Nepal; ^4^Department of Ophthalmology, B.P. Koirala Institute of Health Sciences, Dharan, Nepal

## Abstract

A congenital cystic eyeball is an extremely rare condition, with only 52 cases reported in the literature to date. An orbital cyst replaces the eyeball which occurs due to the complete or partial failure in invagination of the primary optic vesicle during the fourth week of gestation. We discuss a case of a congenital cystic eyeball in a 14-year-old female who presented to us for a cosmetic blemish due to a large swelling in the right eyelid with the absence of a right eyeball since birth. She underwent removal of the cyst followed by an orbital implant and later prosthesis. Diagnosis of the congenital cystic eyeball was made based on the clinical and ultrasound B-scan features, intraoperative findings, and histopathology report. This article adds one more case to the existing literature on the congenital cystic eyeball. Orbital implant with prosthesis after excision of the cyst provided definitive diagnosis and a good cosmetic outcome in our case.

## 1. Introduction

The congenital cystic eyeball (CCE) was first reported by Taylor and Collins in 1906 [1] and later explained in detail by Mann in 1939 [2]. Mann also coined the term “anophthalmos with cyst” for the congenital cystic eyeball [3]. A complete or partial failure in invagination of primary optic vesicle leads to the development of congenital cystic eyeball [4, 5]. Noninvagination of the optic vesicle takes place in the middle of 2 mm and 7 mm phases of the embryonic development which usually occurs during the fourth week of gestation. The cyst which replaces the eye is formed due to the failure of the anterior primary optic vesicle to involute. It should be differentiated from microphthalmos with cyst which occurs due to the failure of fetal fissure closure at 7-14 mm phase, presents as a cyst in the anterior and inferior part of the orbital cavity, and is associated with a microphthalmic colobomatous eye. Cases of CCE are remarkably rare. To date, only 52 cases of congenital cystic eyeball have been reported in the literature [5–12]. We discuss a case of a congenital cystic eyeball in a 14-year female and also describe the clinical features, diagnostic tools, differentials, and the management approach for such cases through an extensive literature review.

## 2. Case Presentation

**Figure 1:**
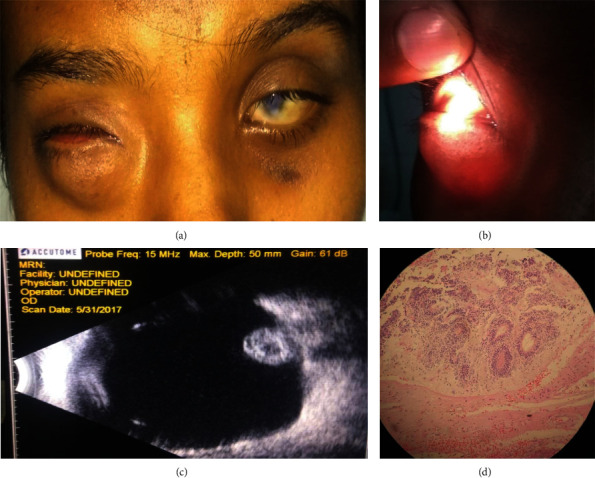
(a) RE congenital cystic eyeball in a 14-year female: LE microcornea with inferior scar. (b) Cyst shows a positive transillumination test. (c) B-scan USG of the RE showing a stump of optic nerve-like structure in the posterior aspect of a large cyst. (d) Histopathology of the excised cyst showing cyst lined by neuroglial tissue. No specific remnants of the eye could be identified.

A 14-year female presented with a complaint of cosmetic disfigurement caused by a large swelling in the right eye (RE) since birth which was gradually increasing with her age. The patient was aware of no vision from that eye. There was no history of perinatal complications or consanguineous marriage. On examination, the vision in RE was no perception of light (NPL) whereas the vision in the left eye (LE) was 1/60 on the Snellen distant visual acuity chart. RE examination revealed a large cystic swelling predominantly in the lower eyelid but no visible eyeball in the palpebral aperture (Figure 1). The swelling was single, soft, smooth, bluish-tinged, nontender, nonpulsatile, and brilliantly transilluminating (Figure 1(b)). The bony orbital rim was normal. Examination of the LE suggested a microcornea, inferior corneal scar, iris coloboma, and chorioretinal coloboma involving the optic disc and macula (Figure 1). The patient underwent B-scan ultrasonography (USG) which demonstrated a large cyst in the right bony orbit with no evidence of a well-formed eyeball. Typically, a stump of the optic nerve-like structure was seen in the posterior aspect of the cyst (Figure 1(c)). A provisional diagnosis of CCE was made based on the clinical and radiological findings. Pediatrician consultation was done, and neuroimaging was performed to rule out systemic associations especially intracranial abnormalities, which was normal. The patient and her parents were counseled regarding the nil visual prognosis and were advised for surgery. The patient had the cyst removed and an orbital ball implant and a conformer placed, and inferior fornix was reconstructed using sutures. The cyst was sent for histopathological examination which revealed an irregular cyst lined externally by a connective tissue layer and internally by glial tissues (Figure 1(d)) without a histologically identifiable eyeball, which confirmed the diagnosis of the congenital cystic eyeball. She was prescribed an ocular prosthesis after 6 weeks of surgery.

## 3. Discussion

A congenital cystic eyeball occurs because of a developmental anomaly occurring during the third week of embryogenesis. An arrest in the invagination of the primary optic vesicles during the 2 mm-7 mm stage is attributed to this rare congenital anomaly [5]. Though the etiology of CCE is unknown, some authors have related it to an inflammatory cause due to the presence of inflammatory cells in the histopathological picture of the cystic eyeball [5].

Cases are usually unilateral, although few bilateral cases have been described in the literature. A case of bilateral congenital cystic eyeball was reported by Sacks and Linderberg. In two cases reported by Hayashi et al., one had bilateral involvement although the nature of the lesion was not established [13]. The patients usually present with a swelling in the eyelid of the involved eye since birth. However, the cyst may not be evident at birth in some cases. Such patients present with an absence of the eyeball and later with a cystic swelling when the cyst progressively enlarges due to the continuous production of fluid into the cyst probably from the neuroglial tissues. This fluid can be dark viscous, serosanguineous, or proteinaceous [13, 14], but the fluids usually have similar biochemical properties as the serum [15].

Diagnostic workup for a case of CCE starts from the examination of the eyes and orbits and extends to the whole body to rule out systemic associations which may be life-threatening. In infants, the cyst can be examined properly using eyelid retractors, such as Desmarre's, to look for the presence of a microphthalmic eye which is the most important differential diagnosis. Where congenital cystic eyeballs have the complete absence of a globe, microphthalmos with cyst usually has a small eyeball and the cyst is attached to the sclera or choroid [11, 16]. Incomplete closure of the fetal cleft leads to microphthalmos with cyst which often has coloboma of uveal tissue, lens, and retina. Cysts are generally placed in the inferior orbit. Inversely, a congenital cystic eyeball typically causes the upper eyelid to bulge, and there is the presence of a pedicle. However, there are some exceptions where the lower eyelid bulges in a congenital cystic eyeball [13, 17]. In our case, the cyst was located in the inferior part of the orbital cavity presenting as a swelling in the lower eyelid.

The fellow eye in our case had microcornea with an inferior corneal scar and, iris, and fundal coloboma. As anophthalmos and microphthalmos (with or without cyst) and uveal coloboma—all are congenital abnormalities occurring due to the failure of invagination of optic vesicle at various stages of development of ocular structures, these are often found in association. Tucker et al. found abnormal second eye in 21% cases of unilateral anophthalmos without a cyst [18]. Although the data with anophthalmos with cyst is limited, we can take reference from the largest case series of anophthalmos with cyst by McClean et al. [19]. In their case series of 34 cases of orbital cysts associated with anophthalmos or microphthalmos, they have described 14 cases of anophthalmos with cyst wherein 5 cases were unilateral with fellow eye normal, 3 cases had microphthalmos in fellow eye with or without uveal coloboma, 3 cases had anophthalmos without a cyst in fellow eye, and the remaining 3 had bilateral anophthalmos with cyst. There were 2 cases described in the case series where the fellow eye had both microphthalmos and uveal coloboma similar to our case. Similarly, Hayashi et al. [13] reported a case of congenital cystic eyeball with microphthalmos in the fellow eye. Hence, our case report is rarer in view of involvement of fellow eye with microphthalmos and uveal coloboma.

Radiological investigations such as B-scan ultrasonography (USG), computed tomography (CT) scan, or magnetic resonance imaging (MRI) form the logical next step in the diagnosis of CCE. B-scan USG is usually readily available at the ophthalmology outpatient department and gives valuable information about the cyst, absence or presence of an eyeball, and an associated optic nerve-like stump. Cysts may be replaced partially or even completely by neuroglial tissues [20]. Baghdassarian et al. found a 2 mm round structure posteriorly, resembling an optic nerve at the posterior aspect of a cyst [21]. We also found a small round stump of an optic nerve-like structure at the posterior part of the cyst in our case on B-scan USG. Studies have revealed the presence of patent optic stalk [22]; however, there have been reports of nonpatent [23] or even absence of a posterior stalk [13]. Reports with CT scan or MRI frequently reveals a cystic mass in the orbital cavity which might be unilateral [24] or bilateral [20, 25]. Microphthalmos and any optic nerve stalks will also be evident on CT or MRI. Usually, extraocular muscles are absent and cystic mass probably has a soft tissue component depending upon the amount of glial proliferation [4, 25].

The mainstay of management of the congenital cystic eyeball is excision of the cyst followed by an orbital implant. Morselli et al. reported a case of the congenital cystic eyeball where they followed up the case serially from 20 weeks of gestation till birth [26]. The case was managed by a multidisciplinary team of ophthalmologists, plastic surgeons, pediatricians, and neurosurgeons. Guthoff et al. reported a congenital cystic eye in a 1-month healthy infant, where during excision of the mass, yellow serous fluid was released [27]. A spherical silicone orbital implant was inserted. The optic nerve was not identified in this study. Holland et al. removed the cyst in a case of the congenital cystic eyeball and replaced it with a bioceramic implant [12]. Our case, an early teenage girl, underwent excision of the orbital cyst with an orbital implant and conformer by an oculofacial plastic surgeon. After 6 weeks, an ocular prosthesis was prescribed for cosmetic rehabilitation.

An irregularly shaped cyst with a connective tissue layer externally and an inner neuroglial tissue layer is the common histopathological picture in CCE. There is no presence of epithelial linings of cysts in the CCE and microphthalmos with cyst, and thus, they are similar in histopathology [28]. However, the absence of a small developed eyeball and the lack of surface ectodermal elements are the main features for differentiating CCE from microphthalmos with a cyst. Our case had features suggestive of CCE without an identifiable eyeball on histopathology.

Although CCE usually does not have associated nonocular abnormalities, some bilateral congenital cystic eyes [17, 29] and unilateral congenital cystic eye [28] with nonocular anomalies have also been reported. Some of these may have intracranial abnormalities such as agenesis of the corpus callosum, midbrain deformity, and basal encephalocele [4, 13]. Furthermore, grey matter heterotopias with corpus callosum agenesis have also been illustrated in MRI [7]. Studies have described the presence of intracranial abnormalities in CCE which required ventriculoperitoneal shunting [4, 11, 25, 29, 30]. Hence, cases of microphthalmia and anophthalmia with or without a cyst need radiological investigation especially neuroimaging to rule out systemic associations such as intracranial abnormalities. Ragge et al. in their review of management of anophthalmia and microphthalmia have described the frequent association with ocular abnormalities and infrequently with nonocular abnormalities such as CHARGE syndrome [31]. Similarly, Das et al. have recently reported a case of congenital cystic eyeball with associated intracranial abnormalities in a 15-day-old girl [32]. A full list of cases reported till date is shown in Table 1 which demonstrates the frequency and the type of the ocular and systemic associations. Our case was reviewed by a pediatrician before the surgery which revealed no neurological abnormality. Studies to date have not demonstrated any hereditary associations or chromosomal defects for the congenital cystic eyeball [4, 13, 17, 30].

Clinical assessment and radiological investigations aid in the confirmation of the diagnosis of CCE. However, a definitive diagnosis can only be made through histopathology. Removal of the cyst followed by an orbital implant, conformer, and later on ocular prosthesis seems to be the appropriate management approach for CCE.

## Figures and Tables

**Table 1:** A table of all 52 cases reported till date with ocular and systemic associations.

S no	Author	Title	Year of publication	No. of cases	Age of onset	Affected eye	Findings		
							RE	LE	Systemic association
1	Rice et al. [33]	Case of congenital cystic Eye and accessory limb of the lower eyelid	1966	One	Eight months	LE	Normal	Cystic swelling which distended the upper lid, while projecting from the left lower lid was a rudimentary accessory limb	Multiple dermal appendages on the face inferotemporal to the left orbit, anterior to the tragus of the ear and an appendage present on the upper part of the neck
2	Dollfus et al. [28]	Congenital cystic eyeball	1968	One	At birth	LE	Normal	A plum sized mass in the left orbit distending the upper lid and projecting between the eyelids).	Malformation of the left nostril and small cutaneous tumors of the left upper lid.
3	Sacks and Lindenberg [29]	Efferent nerve fibers in the anterior visual pathways In bilateral congenital cystic eyeballs	1969	One	6 years	BE	Somewhat firm, spherical mass measuring approximately 10 mm in diameter	Somewhat firm, spherical mass measuring approximately 10 mm in diameter	Severely retarded and had other congenital anomalies consisting of saddle nose, harelip, cleft palate, and bifid left thumb
4	Helveston et al. [23]	Congenital cystic eye	1970	One	One day old	LE	Normal	Mass protruding between the left eye lids	—
5	Baghdassarian et al. [21]	Congenital cystic eye	1973	One	Ten days old	LE	Normal	Cystic mass in the left orbit, bulging forward, stretching the upper eyelid, and displacing the lower eyelid downward	—
6	Waring et al. [30]	Clinicopathologic correlation of microphthalmos with cyst	1976	One	New born	RE	RE cystic eyeball	Normal	Mild microcephaly, severe bilateral cleft lip and palate with absent philtrum; microphallus with left hydrocele; decreased neurologic tone; and hyperconvex fingernails on short stubby fingers
7	Pillai and Sambasivan [25]	Congenital cystic eye-a case report with CT scan	1987	One	Two months	RE	Cystic eye	Normal	—
8	Gupta et al. [34]	Congenital cystic eyeball	1990	One	One day old	LE	Normal	Large cystic mass bulging forwards stretching the upper lid	Microcephaly
9	Pasquale et al. [4]	Congenital cystic eye with multiple ocular and intracranial anomalies	1991	One	New born	LE	Persistent hyperplastic primary vitreous	Left orbital mass protruding through the palpebral fissure	Cerebrocutaneous abnormalities consisting of agenesis of the corpus callosum, midbrain deformity, malformed sphenoid bone, right upper eyelid coloboma, and a left periocular hamartoma
10	Goldberg et al. [17]	Bilateral congenital ocular cysts^∗^	1991	One	One month old	BE	Cystic eye	Cystic eye	Mild facial clefting (median cleft lip and cleft palate) and basal cephalocele
11	Mansour and Li [15]	Congenital cystic eye	1996	One	One day old	RE	Congenital cystic eye	Normal	Holoprosencephaly and tetralogy of Fallot
12	Albernaz et al. [16]	Imaging findings in patients with clinical anophthalmos	1997	One	—	—	Congenital cystic eye	Anophthalmos	1 case out of reported 8 cases had congenital cystic eyeball. Systemic association noted in bilateral anophthalmos cases
12	Hayashi et al. [13]	Congenital cystic eye: report of two cases and review of the literature	1999	Two	Case 1: 13 months Case 2: 2 weeks	Case 1: LE Case 2: LE	Microphthalmos Normal	Cystic eye Cystic eye	—
13	Gupta et al. [20]	Congenital cystic eye with multiple dermal appendages: a case report	2003	One	One day old	LE	Normal	Large orbital mass in the left orbit that bulged forwards and stretched the eyelids	—
14	McClean et al. [19]	The management of orbital cysts associated with congenital microphthalmos and anophthalmos	2003	Fourteen	—	RE: 5 LE: 6 BE: 3	24-month female, left eye cyst right eye microphthalmos, systemically associated with oculo-cerebro-cutaneous syndrome 30-month female, patient had unilateral right eye cyst, no systemic associations 12-month male with both eye cyst and no systemic associations 11-month male with left eye cyst with no systemic association 50-month female with left eye cyst, right microphthalmos with colobomatous iris and retina and developmental delay 3-month female with left eye cyst with no systemic associations 1-month female right eye cyst with cleft lip and palate, atrial septal defect, and choanal atresia 6-month male left eye with no systemic associations 2-month female with right eye cyst and left eye retinal coloboma and no systemic associations 204-month male left eye cyst and right congenital nystagmus 2-month female both eyes cyst with congenital hip dislocation 2 months male both eye cyst with no systemic association 1-month male right eye with fistula-in-ano 2-month male right eye with no systemic association		
15	Robb and Anthony [14]	Congenital cystic eye: recurrence after initial surgical removal	2003	One	Six weeks	RE	Large right orbital cyst, which pushed the upper eyelid forward and filled the interpalpebral space	Normal	—
16	Guthof et al. [27]	Congenital cystic eye	2004	One	4 years	RE	Presented with complete ptotic right upper lid without levator function and displacement of the prosthesis due to the enlarged cystic mass	Normal	—
17	Chaudhry et al. [11]	Congenital cystic eye with intracranial anomalies: a clinicopathologic study	2007	Two	Case 1: 15 days Case 2: six months	Case 1: RE Case 2: LE	Case 1: RE cyst Case 2: high myopia in RE	LE anophthalmic socket without cyst LE cyst	Both cases had intracranial abnormalities requiring ventroperitoneal shunt and one case had hemifacial ipsilateral hypotony
18	Gupta et al. [7]	Congenital cystic eye: Features on MRI	2007	One	5 years	LE	Normal	Presented with a left orbital mass	—
19	Quintyn-Ranty et al. [35]	Congenital cystic eye	2007	One	Neonatal	LE	Normal	Tomodensitometry revealed a solid, cystic orbital mass, with no calcification or bone lysis	—
20	Kavanagh et al. [8]	Detection of a congenital cystic eyeball by prenatal ultrasound in a newborn with Turner's syndrome	2007	One	At birth	RE	Presented with RE anophthalmos with cyst	Normal	Turner syndrome
21	Subramaniam et al. [36]	Prepucial skin graft for forniceal and socket reconstruction in complete cryptophthalmos with congenital cystic eye	2008	One	23 days old	RE	Complete cryptophthalmos and congenital cystic eye	Normal	—
22	Gangadhar et al. [6]	Congenital cystic eye with meningocele	2009	One	2 years s	RE	RE cyst	Normal	Meningocoele
23	Mehta et al. [37]	Congenital cystic eye: a clinicopathologic study	2010	One	13 years	LE	Normal	A mass bulging through the ptotic left upper eyelid On opening the palpebral aperture, a white sclera like structure could be seen beneath the conjunctiva with absence of other anterior segment structures	Associated with ectopic glial tissue in the brain
24	Tsitouridis et al. [10]	Congenital cystic eye with multiple dermal appendages and intracranial congenital anomalies	2010	One	3 months	LE	Normal	Mass in the left orbit and the dermal appendages on the ipsilateral side of the face	—
25	Morselli et al. [26]	Congenital cystic eye: from prenatal diagnosis to therapeutic management and surgical treatment	2011	One	20-week gestation female fetus	RE	Congenital cystic eye	Coloboma and corneal dermoid	Left brachycephaly
26	Pinto et al. [38]	Congenital cystic eye with corpus callosum hypoplasia: MRI findings	2011	One	3 months	LE	Normal	Cystic eye	Corpus callosum hypoplasia
27	Doganay et al. [39]	Bilateral congenital cystic eye posterior to the lower eyelid: case report	2012	One	One day old	BE	Cystic eye	Cystic eye	—
28	Singer et al. [9]	Congenital cystic eye in utero: novel prenatal magnetic resonance imaging findings	2013	One	26-week gestation	LE	Normal	Cystic eye	Left frontal dysplasia, colpocephaly, and agenesis of the corpus callosum and septum pellucidum discovered in utero via ultrasonography
29	Cefalo et al. [40]	Congenital cystic eye associated with a low-grade cerebellar lesion that spontaneously regressed	2014	One	6 month old	LE	Normal	Cystic eye	Cerebellar lesion accidentally detected at magnetic resonance imaging
30	Holland et al. [12]	Congenital cystic eye with optic nerve	2015	One	3 days old	RE	Cystic eye	Normal	—
31	Souhail et al. [41]	Congenital cyst eye, one clinical case	2015	One	7 years	RE	Fleshy mass in the right eye, the upper lid appeared ballooned and a reddish pink mass was bulging out	Normal	—
32	Yan et al. [42]	Rare orbital cystic lesions in children	2015	One	6 months	LE	Normal	Ptosis and protrusion of left upper eyelid. A large well-defined soft mass	Complete agenesis of the corpus callosum, hydrocephaly, and asymmetry of the ventricular system
33	Musa et al. [43]	Congenital cystic eye: a clinicopathological review	2018	One	7 months	RE	Cystic eye	Normal	—
34	Stahnke et al. [44]	Management of congenital clinical anophthalmos with orbital cyst: a Kinshasa case report	2018	One	14 months	LE	Normal	A transilluminating cyst protruding out of the left orbit	—
35	Harakuni et al. [45]	A rare case of left sided anophthalmos with congenital cystic eyeball with right sided microphthalmos	2019	One	19 years	LE	Microphthalmos, with horizontal nystagmus and healed perforated corneal ulcer	Anophthalmos	—
36	Das et al. [32]	Congenital cystic eyeball with intracranial anomalies: a rare entity	2021	One	15 days	LE	Normal	Cystic eye	Dysgenesis of corpus callosum with dorsal interhemispheric cyst communicating with the third ventricle

## Data Availability

No datasets were generated or analyzed during the current study.
